# Probing the Residual Structure in Avian Prion Hexarepeats by CD, NMR and MD Techniques

**DOI:** 10.3390/molecules180911467

**Published:** 2013-09-16

**Authors:** Luigi Russo, Luca Raiola, Maria Anna Campitiello, Antonio Magrì, Roberto Fattorusso, Gaetano Malgieri, Giuseppe Pappalardo, Diego La Mendola, Carla Isernia

**Affiliations:** 1 Dipartimento di Scienze e Tecnologie Ambientali, Biologiche e Farmaceutiche, Seconda Università di Napoli, via Vivaldi 43, 81100 Caserta, Italy; E-Mails: luigi.russo2@unina2.it (L.R.); lucaraiola@gmail.com (L.R.); mari_camp@libero.it (M.A.C.); roberto.fattorusso@unina2.it (R.F.); gaetano.malgieri@unina2.it (G.M.); 2 CNR-Istituto di Biostrutture e Bioimmagini, viale Doria 6, 95125 Catania, Italy; E-Mails: leotony@unict.it (A.M.); pappalardo.cnr@unict.it (G.P.); 3 Centro Interuniversitario di Ricerca sui Peptidi Bioattivi, Università di Napoli “Federico II”, Via Mezzocannone 16, 80134 Napoli, Italy; 4 Dipartimento di Farmacia, Università di Pisa, Via Bonanno Pisano 6, 56126 Pisa, Italy; E-Mail: diego.lamendola@farm.unipi.it

**Keywords:** avian prion repeats, NMR, CD, conformational ensemble, polyproline II

## Abstract

Many proteins perform essential biological functions by means of regions that lacking specific organized structure exist as an ensemble of interconverting transient conformers. The characterization of such regions, including the description of their structural propensities, number of conformations and relative populations can provide useful insights. Prion diseases result from the conversion of a normal glycoprotein into a misfolded pathogenic isoform. The structures of mammal and chicken prion proteins show a similar fold with a globular domain and a flexible N-terminal portion that contains different repeated regions: octarepeats (PHGGGWGQ) in mammals and hexarepeats (PHNPGY) in chickens. The higher number of prolines in the hexarepeat region suggests that this region may retain a significant amount of residual secondary structure. Here, we report the CD, NMR and MD characterization of a peptide (2-HexaPY) composed of two hexarepeats. We combine experimental NMR data and MD to investigate at atomic level its ensemble-averaged structural properties, demonstrating how each residue of both repeats has a different quantified PPII propensity that shows a periodicity along the sequence. This feature explains the absence of cooperativity to stabilize a PPII conformation. Nonetheless, such residual structure can play a role in nucleating local structural transitions as well as modulating intra-molecular or inter-molecular interactions.

## 1. Introduction

Many proteins perform crucial functions by means of disordered regions. Structural disorder under physiological conditions offers a variety of functional advantages: flexibility of interaction with different partners, specific but low-affinity binding, and fine modulation by post-translational modifications. The structural characterization of such regions can therefore provide useful information regarding the role of secondary structure propensities in directing essential biological functions. The secondary structure adopted by a protein is generally driven by local dihedrals associated to a particular aminoacid: the presence of a given residue is not a random choice of nature, but it is intrinsically related to the flexibility *versus* the stiffness of a protein. The extended flexible disordered N-terminal tail of the prion protein, whose C-terminal portion shows a globular structured domain, has captured the attention of many studies.

NMR studies have established the structure of mammal, chicken, turtle and frog prion proteins, showing closely similar fold of the globular domains, containing three α-helices, one short 3_10_-helix and a short antiparallel β-sheet [1]. The low solubility of PrP even without GPI anchor and its strong propensity to aggregate has required that most of the structural studies were performed at low pH and mainly with N-terminally truncated variants.

The chicken PrP^C^, despite only 30% of identity in the primary sequence with its mammal counterpart, maintains the presence of tandem amino acid repeats in the N-terminal region (PHNPGY in avian, PHGGGWGQ in mammals), followed by a highly conserved hydrophobic core.

The repeat region of the avian prion protein suggests some important differences when compared with the human corresponding region. In particular, the normal isoform of mammalian prion protein is totally degraded by proteinase K while the chicken prion protein is not, thereby producing, from the N-terminal domain, peptide fragments stable to further proteolysis [2]. The mammalian octarepeat region contains 50% of glycine and 12% of proline residues, while avian hexarepeat accounts for 16% and 33%, respectively. The higher amount of glycine in the human protein is in agreement with the flexible and disordered structure of the N-terminal domain, while the increased content of prolines suggests the presence of a preferred structured conformation of this domain in the avian species [3]. Accordingly, CD data reported for the entire protein or different peptide fragments containing the hexarepeat sequence clearly suggest the presence of more than one conformation and not simply a random coil one [2,4,5]. The location of the PXXP motif along the peptide backbone appears to be important to determine the conformational preferences [5]. The presence of PXXP motif is often associated with the formation of an extended poly-L-proline II (PPII) structure or turns [6,7,8]. The sequence of the avian hexarepeat can be reported as PHNPGY which reveals the presence of two different PXXP motifs (*i.e.*, PHNP and PGYP) along the whole tandem repeat domain. Previous studies described the conformational behaviour of the Ac-PHNPGY-NH2 peptide [9]. It was found that the Asn-Pro residues populated dihedral angles typical of a PPII conformation, while a turn-like structure in the NPGY region was preferred. Further studies on the tetraHexaPY (PHNPGY)_4_ conformation, at different pH values [10], have shown for this peptide a periodic-like shape that reflects the periodicity in the primary structure and with it the differences with the flexible and disordered structure of the human N-terminal domain.

Therefore, in order to fully explore the conformational preferences of the chicken hexarepeat region, here, we report the CD, NMR and MD characterization of a dodecapeptide Ac-PHNPGYPHNPGY-NH_2_ (2-HexaPY) composed of two chicken hexarepeats. We investigate at an atomic level its ensemble-averaged structural properties using an approach that combines experimental NMR data and MD. Our data strongly support that, despite the reported high flexibility of the hexarepeat region, the 2-HexaPY is not completely random coil in solution but rather shows a residue- based (per residue) quantified propensity to poly-l-proline II conformation. Our findings are in agreement with the reported CD behaviour of tetraHexaPY [10] offering valuable hints on PPII conformation propensity of the full hexarepeat region of the chicken prion protein.

## 2. Results and Discussion

### 2.1. Circular Dichroism Measurements

Due to their high flexibility, short peptides generally present a random coil structure. However the PXXP motif in the avian prion protein suggests that this region may possess a defined secondary structure [11].

**Figure 1:**
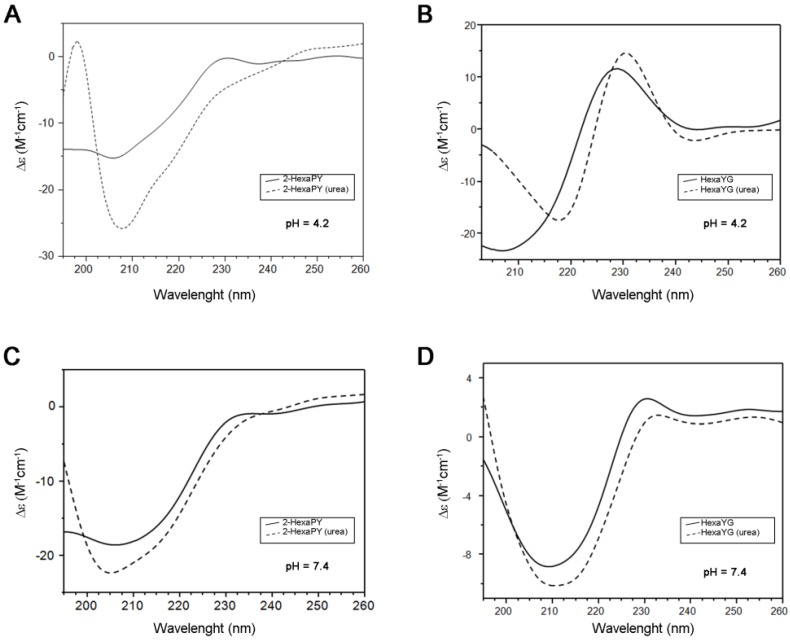
CD spectra of 2-HexaPY (**A**,**C**) and HexaYG (**B**,**D**) in H_2_O (solid line) and 1 × 10^−3^ M Urea (dotted line). Spectra were recorded at pH 4.2 (**A**,**B**) and pH 7.4 (**C**,**D**) for both peptides.

The CD spectrum of 2-HexaPY in aqueous solutions at acidic pH is characterized by a strong negative minimum at 205 nm and a weaker positive band centered at 230 nm (Figure 1A). The spectrum shape is similar to that previously reported for the tetrahexarepeat (tetraHexaPY) [10] and suggests the presence of a polyproII (PPII) helix conformation. To verify this hypothesis, CD spectra have been carried out also in the presence of 1 mM urea, that is well known to induce peptide unfolding. The CD spectrum in urea solution (Figure 1A) shows a decrease of maximum at 230 nm and a blu-shift of minimum at 205 nm, indicating a decrease of polypro(II) content toward a random coil conformation.

The CD spectra deconvolution by means of CDPro software package containing three different algorithms [12,13] indicated that in aqueous solution, the 2-HexaPY displays a 11% content of polypro(II), that decreases to 2% in presence of urea. In order to evaluate the sequence effect on the PPII propensity, the same spectra were recorded on one of the possible hexapeptides encompassing the sequence of the repeat region. The chosen peptide was HexaYG (Ac-YPHNPG-NH_2_) and its spectra are shown in Figure 1B. The reason for choosing HexaYG among the other possibilities will be clarified in the next section.

The CD spectra of HexaYG recorded in aqueous solutions are characterized by an intense negative band between 200 and 210 nm along with a positive band between 228 and 232 nm. In urea, the band at 230 nm is shifted and increases its intensity. The CD spectra deconvolution gives a 2.6% of polyproII content for HexaYG in aqueous solution. In presence of urea the polypro(II) content for HexaYG remains unchanged.

As PPII conformation is known to be favoured in the acidic pH range, CD spectra of peptides were carried out also at physiological pH both in urea and in water (Figure 1C,D). The CD spectra of 2-HexaPY and HexaYG in aqueous solutions at pH 7.4 show a decrease of the maximum at 230 nm confirming a pH dependent decrease of PPII content, likely due to the histidine deprotonation (Figure 1C and Figure 1D). The CD spectra deconvolution confirms that at physiological pH the content of polypro(II) conformation is 5.3%.

### 2.2. Nuclear Magnetic Resonance Assignments

The CD results indicate 2-HexaPY as a suitable model to explore the conformational heterogeneity of the full hexarepeat region of the chicken prion protein. The solution behaviour at pH 4.2 of this peptide offers the possibility to obtain an accurate interpretation of NMR spectra in terms of chemical shift assignments and NOEs evaluation.

At 300 K the peptide 2-HexaPY shows a well resolved 1D spectrum, characterized by a good dispersion of the proton resonances. In the amide region two major species which slowly interconvert on the NMR timescale are clearly visible. They are approximately populated in a ratio of 1 to 4 and represent two of the possible cis-trans isomers of the Xxx-Pro peptide bonds; only the major conformer gives detectable NOE cross peaks. The other conformers are also present in solution and account for about 5% of the total population.

The sequential assignment of all the major conformer protons of 2-HexaPY was performed using the standard strategy described by Wuthrich [14]. TOCSY [15] experiment was used to identify spin systems, DQFCOSY [16] spectrum was analyzed to measure three-bond spin-spin coupling constants, NOESY [17] and ROESY [18] were analyzed to obtain inter-residue connectivities and to distinguish equivalent spin systems. The complete ^1^H-NMR assignment is summarized in the Table 1. To explore the solution conformations of this peptide, proton chemical shifts, scalar coupling constants ^3^*J*_HNH__α_ and ROE pattern were investigated.

**Table 1:** ^1^H-NMR resonances assignment (ppm) for 2-HexaPY in H_2_O (300 K, pH 4.2).

	H^N^	*α*H	*β*H	others
PRO		4.18	2.10, 1.67	*γ*CH2 1.81 *δ*CH2 3.48
HIS	8.47	4.57	3.11, 3.02	*δ*CH 7.12 *ε*CH 8.46
ASN	8.34	4.83	2.69, 2.56	*γ*NH_2_ 7.47, 6.81
PRO		4.29	2.13, 1.80	*γ*CH2 1.88 *δ*CH2 3.64, 3.60
GLY	8.21	3.73		
TYR	7.81	4.69	2.94, 2.78	*δ*CH 6.93 *ε*CH 6.69
PRO		4.24	2.09, 1.68	*γ*CH2 1.81 *δ*CH2 3.62, 3.32
HIS	8.36	4.55	3.11, 3.03	*δ*CH 7.15 *ε*CH 8.47
ASN	8.38	4.83	2.68, 2.54	*γ*NH_2_ 7.49, 6.82
PRO		4.31	2.13, 1.79	*γ*CH2 1.88 *δ*CH2 3.65, 3.61
GLY	8.28	3.78, 3.72		
TYR	7.79	4.39	2.96, 2.85	*δ*CH 7.00 *ε*CH 6.70

### 2.3. Chemical Shift Analysis

**Figure 2:**
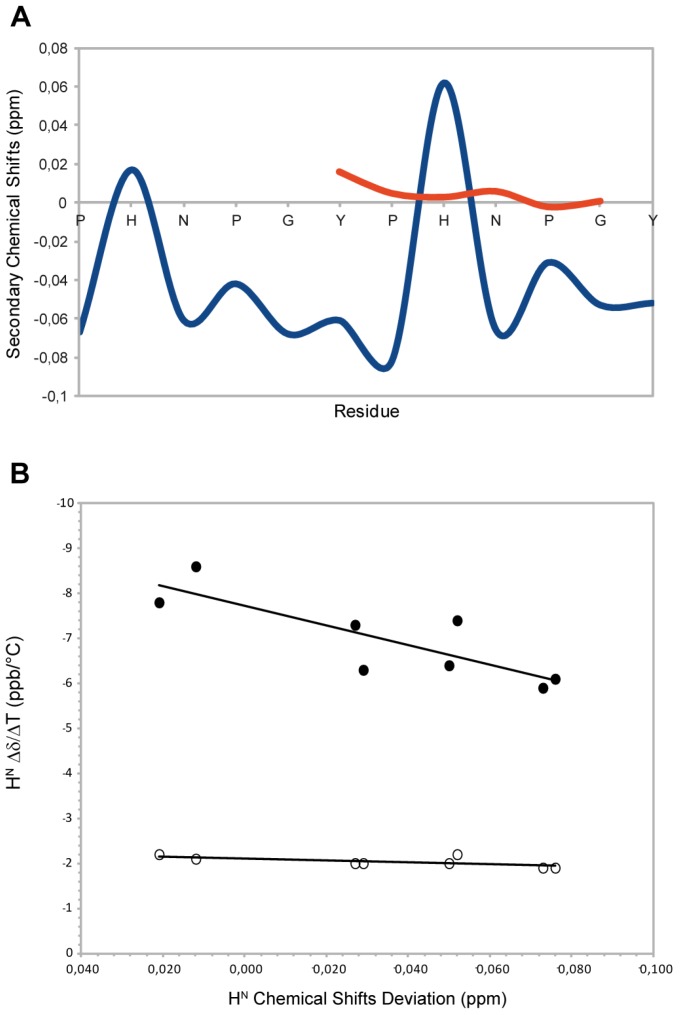
(**A**) Secondary Chemical Shifts of H_α_ obtained by subtraction of intrincic statistical coil shifts recorded in 8 M urea for 2-HexaPY (Blue) and HexaYG (orange). (**B**) Amide H_N_ Chemical Shift Deviation (CSD)–Δδ/ΔΤ correlation for 2-HexaPY (filled circles). The open circles shown define the condition for H^N^s involved in H-bond reported by Andersen and co-workers [25].

Chemical shifts are sensitive reporters of dihedral angles of peptide backbones and therefore of the secondary structure content [19,20,21]. In particular, since well-defined chemical shift changes are associated with α-helices and extended β-strands [21,22], deviations of αH chemical shifts from random or statistical coil values provide information about peptide secondary structure. To get suitable “statistical coil” reference chemical shifts, the NMR resonances of 2-HexaPY were also assigned in 8 M urea [23] at 300 K and were used as intrinsic statistical coil shifts (δisc). ‘Secondary shifts’ (deviations from random coil values) were calculated as δ–δisc. This referencing method allows to have an evaluation of the secondary shifts with less noise than the traditional method based on chemical shifts databases. As reported in the Figure 2A, the residues N,P,G,Y and P show negative Hα secondary shifts (average upfield shift −0.04 ppm) suggesting that the peptide populates conformations with structural proprieties different from the random coil condition. This is mostly due to prolines that sterically restrict the conformational space. These chemical shift deviations are consistent with the typical values observed for the PPII helix conformation (ΔδHα= −0.02ppm) [24] (Figure 1A). The histidine residues show a positive secondary shift that is more evident for the second histidine. These residues interrupt the PPII helix propensity and the effect is more marked when the His is located at the centre of the peptide sequence. To further confirm the structural propensity observed, we decided to investigate a component shorter peptide named HexaYG (Ac-YPHNPG-NH_2_) in which the histidine is in the middle of the sequence. Interestingly the secondary chemical shifts analysis (Figure 2A) indicates that this peptide adopts a completely random coil conformations in agreement with the results obtained by the deconvolution of CD data.

### 2.4. Temperature Coefficients

The high temperature coefficients for all amide protons (Figure 1B), indicate that the backbone NH groups are exposed to the solvent and not involved in any intramolecular hydrogen bond interaction. In addition, the correlation between the backbone amide H_N_ chemical shift temperature gradients (Δδ/ΔT) with the deviation of the H_N_ protons from 2-HexaPY statistical-coil reference chemical shift (ΔδH_N_) (Figure 1B), proposed by Andersen *et al.* [25], confirms that no persistent hydrogen bond are present in the 2-HexaPY peptide.

#### 2.5. ^3^JHNHα Coupling Constants

The torsion angles φ and Ψ are key parameters for defining the backbone conformation of a polypeptide chain [26]. In solution these angles can be detected experimentally by NMR via spin-spin coupling constants, which in case of ^3^*J* coupling constants can be related to specific torsion angles by Karplus relationships [27,28]. Three bond ^3^*J*_HNHα_ coupling constants for extended conformations are between 6–9 Hz, whereas smaller values (2–5 Hz) are commonly observed for α helix [14]. However, when multiple conformations are adopted, and there is rapid inter-conversion between them, the experimental coupling constants will be averaged over the contributing conformers [29]. As result, the interpretation of coupling constants regarding specific conformation requires caution. 

**Figure 3:**
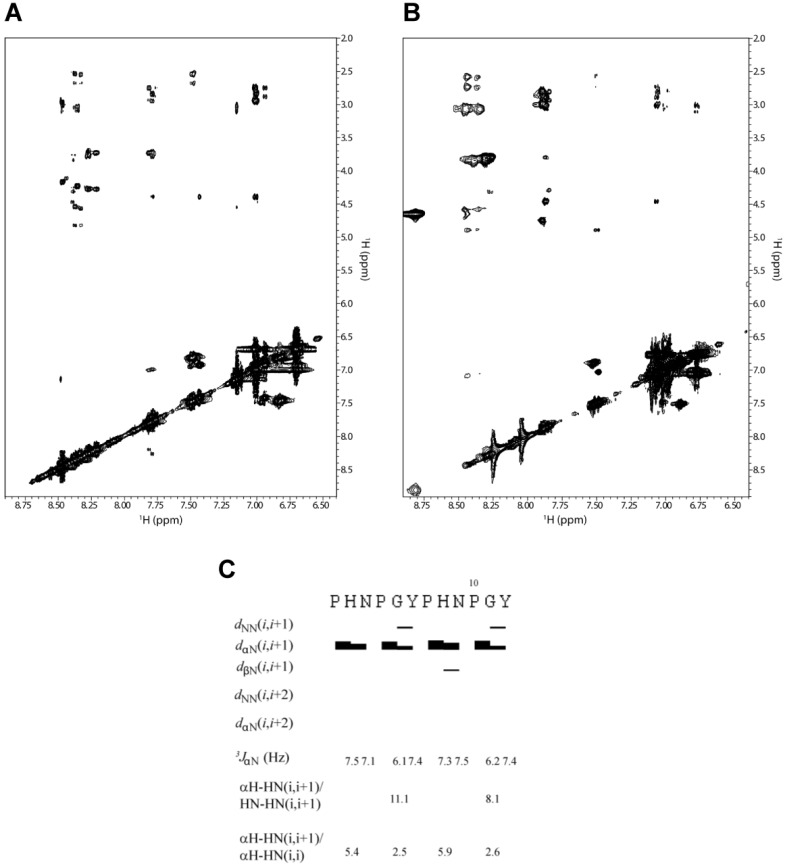
The amide proton region of the 2D ^1^H-^1^H ROESY spectrum of 2-HexaPY acquired in water (**A**) and urea (**B**), respectively. (**C**) Summary of NMR parameters of 2-HexaPY. The summary includes the NOEs diagram, ^3^JHNHα coupling constants at 300 K and temperature coefficients. The intensity ratios of αH–HN(i,i+1)/HN-HN(i,i+1) and αH–H N(i,i+1)/αH–HN(i,i) are also reported.

The coupling constants measured for 2-HexaPY peptide are summarized in Figure 3C and were found to be in the 6.0 to 7.5 Hz range. Additionally, the average <φ> backbone dihedral angles derived from Karplus equation [28], via the equation given by Vuister and Bax [30], were in the ranging from −82° to −85°. Altogether our data are in accordance with a relatively flexible backbone and/or PPII conformations.

### 2.6. NOE Evaluation

The intensity ratio between sequential αH–HN (i, i+1) and HN-HN (i, i+1) NOEs is a useful measure for secondary structure, since it depends on the dihedral angles (φ and ψ). Using a population-weighted random coil model, Dobson and co-workers predicted that the intensity ratio of αH–HN (i, i+1)/HN-HN (i, i+1) is about 1.4 for random coil structure. This value is markedly different from the ratio estimated for extended β-strand conformation (~55) [31]. We performed an analysis of NOE intensities for the 2-HexaPY peptide using ROESY spectrum (Figure 3A and Figure 3B) as no signals were observed in the NOESY spectrum due to the molecular tumbling of the peptide in solution. As expected, the spectrum does not show any long range NOEs, suggesting that the peptide does not adopt a compact structure. Moreover the HN-HN (i, i+1) NOEs were not detected for any residues of the peptide with the exception of a weak contact observed between Gly and Tyr of each repeat (Figure 3C). Additionally, αH–HN (i, i+1) cross-peaks were medium/strong. These data result in a high αH–HN (i, i+1)/HN-HN (i, i+1) intensity ratio, which is consistent with a predominantly extended rather than random coil conformation.

The NOE intensity ratio αH-HN (i, i+1)/αH-HN (i, i) provided further evidence for an extended conformation in solution. A ~2.3 value is predicted for the population-weight random coil, whereas values > 4 are predicted for β-strands and values > 2.3 are predicted for PPII helical conformations. As reported in Figure 3C the intensity ratio αH-HN (i, i+1)/αH-HN (i, i) suggest that 2-HexaPY is not completely random coil but shows a significant propensity to PPII conformations.

An important structural characteristic of the PPII helix is that the peptide bonds preceding proline residues adopt trans configurations. To distinguish between cis/trans configuration of the imide bonds in the 2-HexaPY peptide, the inter-proton distances between the Hα of the preceding residues and the proline Hα and Hδ were measured [32]. A short distance with a strong NOE signal between Hα of a preceding residue and Hδ of a proline is diagnostic for the trans configuration, whereas a short distance between Hα of a preceding residue and Hα of a proline is diagnostic for the cis configuration. The ROESY spectrum displays for both prolines of each reapeat a strong Hα/Hδ(i, i+1) cross peak indicating trans configurations further confirming that the peptide in solution has a significant propensity to occupy the PPII helical conformation.

### 2.7. Hydrodynamic Proprieties

A full description of intrinsically disordered or unfolded state, requires information about the ensemble distribution of molecular size, shapes and the extent of compactness. Diffusion parameters can be related directly to an effective hydrodynamic radius in according to the Stoke-Einstein equation and can thus be used to study molecular size. Therefore, using the average translational diffusion coefficient, hydrodynamic radii of Rh = 9.9 ± 0.2 Å and Rh = 11.0 ± 0.2 Å have been obtained for the 2-HexaPY peptide in water and urea, respectively. The hydrodynamic radii clearly indicate that the peptide in solution samples more compact than random coil conformations.

### 2.8. Conformational Ensemble of 2-HexaPY

Different disordered states, including unfolded, molten globule and intrinsically disordered states, exhibit wide variability of compactness and transient or residual structure. Therefore, unlike a folded protein, which can typically be represented as a single structure or as an ensemble of closely related structures, the high degree of conformational dynamics of a disordered state needs for its representation an ensemble of fairly heterogeneous conformers.

To describe the structural ensemble of 2-HexaPY, we applied a computational approach based on experimental NMR data (see Experimental). We have used ENSEMBLE [33,34,35] as computational software and chemical shifts, distance restraints (Supplementary Table S1) and ^3^*J*_HNH__α_ coupling constants as conformational constraints. These experimental restraint types account for different structural proprieties including also secondary and tertiary structure. We generated a pool of 50,000 conformations and selected a final ensemble of 100 structures that are collectively consistent with the experimental NMR data. Additionally, we generated a reference “coil” ensemble using the software TraDES [36]. The average hydrodynamic radius of the “coil” ensemble was estimated with the HYDROPRO program [37,38,39]. According to the hydrodynamic proprieties, the random coil ensemble average hydrodynamic radius (Rh = 11.2 ± 0.5 Å) is in a good agreement with the data obtained for the 2-HexaPY in urea (Rh = 11.0 ± 0.2 Å).

**Figure 4:**
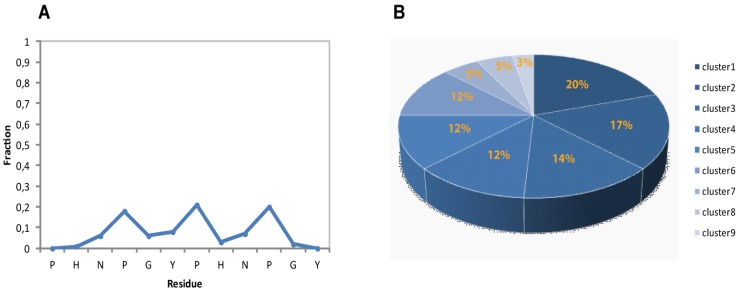
(**A**) Secondary structural propensity of 2-HexaPY Ensemble: fraction of conformers in the PPII region of the Ramachandran plot as a function of the residue number. (**B**) Cluster analysis of the final Ensemble.

The residue-specific secondary structure content, considering the fraction of residues within PPII region of Ramachandran space [40,41], was analyzed by STRIDE software [42]. In Figure 4A is reported the residue-specific population of PPII helical conformation for the calculated ensemble. Overall the 2-HexaPY peptide shows a moderate amount of residual secondary structure (Figure 4A) In particular, each residue of the region NPGYP of both repeat has a different PPII secondary structural propensity. This feature indicates the absence of a cooperativity necessary for a PPII. The maximum value is the one estimated for prolines with a 20% population.

Considering that we have characterized the major conformer and also that in solution minor conformations are present, the obtained PPII fraction (14%) is in good agreement with the PPII population content estimated by CD data (11%).

When the dynamics or motions are extensive, conformational families are believed to be a more realistic representation of the structure [43,44,45,46]. For these reason, in order to visualize the structural diversity induced by the conformational flexibility of the 2-HexaPY peptide, we extracted using the program NMRCLUST [43], the conformationally related subfamilies of structures.

**Figure 5:**
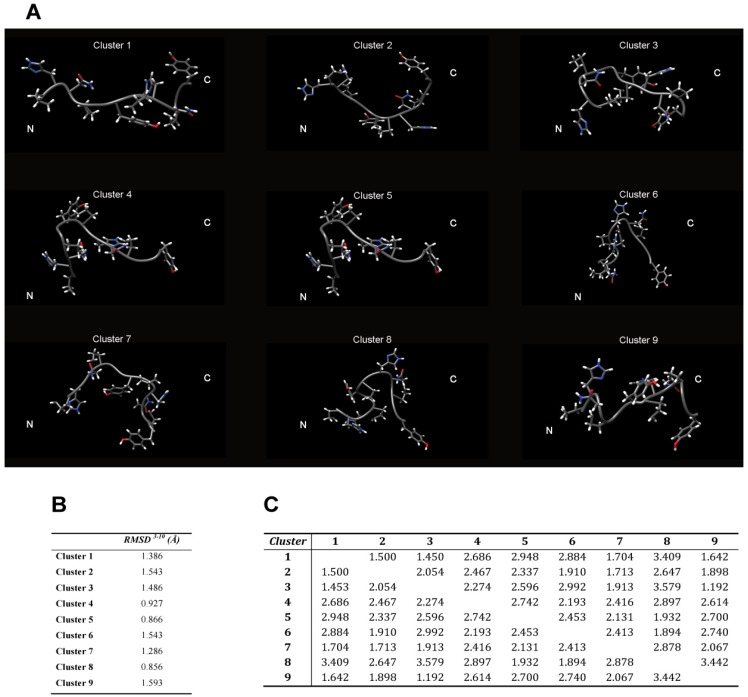
(**A**) The representative conformer from the final Ensemble of 2-HexaPY for each of the nine clusters based on backbone heavy atoms RMSD. The root mean square distributions for backbone heavy atoms of the residues 3–10 with respect to the mean coordinate position within (**B**) and between (**C**) each cluster is also reported.

The backbone heavy atoms of residues 3–10, the region showing the secondary structure propensity, were superimposed. The ensemble was clustered on the backbone heavy atoms of residues 1–12. In total 9 subfamilies of structures were found (Figure 4B and Figure 5A). Among these, the first six contain 87 of the 100 structures, whereas each of the other classes contained five or fewer structures. The conformers in each subfamily are moderately well defined over the entire amino acid sequence. The root mean square distributions for backbone atoms of residues spanning from Asn3 to Pro10 with respect to the mean coordinate position range from 0.9 Å to 1.6 Å (Figure 5B). However, the first 6 subfamilies of structures are equally populated (Figure 4B) indicating the 2-HexaPY displays an high degree of conformational flexibility. Finally, a comparison between the mean structure of each subfamily (Figure 5A) outlines the conformational differences among them. In particular the resulting distribution of RMSD values reported in Figure 5B and Figure 5C indicate the high structural heterogeneity within and between each cluster of the ensemble that strongly depends on the intrinsic flexibility of the peptide in solution. Overall the data suggest that the peptide 2-HexaPY samples a wide conformational space with a moderate propensity for PPII helix conformation.

### 2.9. Validation of the Ensemble

One strategy for assessing the accuracy of calculated ensemble conformers is the cross-validation. We performed a cross-validation analysis for 2-HexaPY ensemble using the hydrodynamic proprieties that were not considered in the ensemble generation. In particular, we back-calculated for each conformer of the ensemble the hydrodynamic radius using the HYDROPRO software [37,38,39]. A comparison of the calculated average hydrodynamic radius (Table 2) (Rh = 10.1 ± 0.4 Å) with the experimental value (Rh = 9.9 ± 0.2 Å) indicate that the calculated ensemble can properly describe the experimental data observed in solution. Moreover, comparing the hydrodynamic properties of the ensemble obtained for 2-HexaPY peptide with those back-calculated for the “coil” statistical ensemble, we further confirmed the accuracy of the ensemble generated from our NMR experimental data.

**Table 2:** Comparison of the experimental hydrodynamic radius estimated in water and urea with the values back-calculated for the obtained ensemble of 2-HexaPY(a) and the TraDES random coil ensemble (b)

	Experimental (water)	Calculated	Experimental (urea)	Calculated (Coil) ^b^
		(2-HexaPY) ^a^		
Hydrodynamic radius (Å)	9.9 ± 0.2	10.1 ± 0.4	11.0 ± 0.2	11.2 ± 0.5

## 3. Experimental

### 3.1. General

All N-fluorenylmethoxycarbonyl (Fmoc)-protected amino-acids, (Fmoc-Gly-OH, Fmoc-Pro-OH, Fmoc-Asn(Trt)-OH, Fmoc-His(Trt)-OH, Fmoc-Tyr(tbu)-OH and 2-(1-H-benzotriazole-1yl)-1,1,3,3-tetramethyluronium tetrafluoroborate (TBTU), were obtained from Novabiochem (Laufelfingen, Switzerland)); Fmoc PAL-PEG resin, N,N-Diisopropylethylamine (DIEA), N,N-dimethylformamide (DMF, peptide synthesis grade) and 20% piperidine-DMF solution were from Applied Biosystems (Foster, CA ,USA); N-hydroxybenzotriazole (HOBT), triisopropylsilane (TIS), trifluoroacetic acid (TFA), ethandithiol (EDT), were purchased from Sigma-Aldrich (St. Louis, MO, USA). All other chemicals were of the highest available grade of purity and were used without further purification. Preparative reversed-phase high-performance liquid chromatography (rp-HPLC) was carried out by means of a Varian PrepStar 200 model SD-1 chromatography system equipped with a Prostar photodiode array detector with detection at 222 nm. Purification was performed by eluting with solvent A (0.1% TFA in water) and B (0.1% TFA in acetonitrile) on a Vydac C18 250 × 22 mm (300 Å pore size, 10–15 µm particle size) column, at a flow rate of 10 mL/min. Analytical rp-HPLC analyses were performed using a Waters 1525 instrument, equipped with a Waters 2996 photodiode array detector with detection at 222 nm. The peptide samples were analysed using gradient elution with solvent A and B on a Vydac C18 250 × 4.6 mm (300 Å pore size, 5 µm particle size) column, at a flow rate of 1 mL/min.

### 3.2. Peptide Synthesis and Purification

The synthesis and the purification of the dodecapeptide Ac-(PHNPGY)_2_-NH_2_ (2-HexaPY) has been reported elsewhere [5]. The Hexapeptide Ac-YPHNPG-NH_2_ (HexaYG) was synthesized in the N-acetylated and C-amidated form to avoid end group effects and to mimic more properly their protein fragment character. It was assembled using the solid phase peptide synthesis strategy on a Pioneer^TM^ Peptide Synthesiser. All residues were introduced according to TBTU/HOBT/DIEA activation method for Fmoc chemistry on Fmoc-PAL-PEG resin (substitution 0.22 mmol/g, 0.33 mmol scale synthesis, 1.5 g of resin. Removal of Fmoc protection during synthesis was achieved by means of 20% piperidine solution in DMF. N-terminal acetylation was performed by treating the fully assembled and protected peptide resins (after removal of the N-terminal Fmoc group) with a solution containing Acetic anhydride (6% *v/v*) and DIEA (5% *v/v*) in DMF. The peptide was cleaved off from the respective resin and simultaneously deprotected by treatment with a mixture of TFA/TIS/H2O (95/2.5/2.5 *v/v*) for 2 h at room temperature. The solution containing the free peptide was filtered off from the resin and concentrated *in vacuo* at 30 °C. The peptide was precipitated with cold freshly distilled diethyl ether. The precipitate was then filtered, dried under vacuum, re-dissolved in water and lyophilised. The resulting crude peptide was purified by applying preparative rp-HPLC. The peptide was eluted with the following protocol: from 0 to 8 min isocratic in A, then linear gradient from 0 to 5% B over 15 min, finally isocratic gradient in 5% B from 15 to 25 min. Hexapeptides HexaYG: Mass calculated for C33H44N10O9 M = 724.33, *m/z* = 724.78 ESI-MS [Obsd *m/z*: (M+H)^+^ 725.5].

### 3.3. CD Measurements

CD spectra were recorded on a JASCO 810 spectropolarimeter at a scan rate of 50 nm min^−1^ and 0.1 nm resolution. The path lengths were 1 cm, in the 190–260 nm range. The spectra were recorded as an average of 20 scans. The CD instrument was calibrated with ammonium(+)-camphor-10-sulfonate. Peptide solutions were freshly prepared in water and in urea 1 × 10^−3^ M solution, in a concentration range of 1 × 10^−5^–1 × 10^−6^ M. The pH was varied by addition of potassium hydroxide or sulphuric acid in water solutions. CD spectra of peptide in Urea solution have been obtained by subtracting urea signal at 1 × 10^−3^ M. CD spectra were analyzed and deconvoluted by means of three different algorithms (CONTIN, SELCON3, and CDSSTR) [12,13].

### 3.4. NMR Experiments

All ^1^H-NMR experiments were carried out at 500 MHz using a Varian Unity 500 spectrometer located at the Dipartimento di Scienze e Tecnologie Ambientali, Biologiche e Farmaceutiche in Caserta (Italy). The lyophilized peptides were rehydrated in H_2_O/D_2_O 90/10 (*v/v*) and the pH of aqueous solutions was adjusted at 4.2. An identical sample containing 8M urea was used to define the reference “intrinsic statistical coil” chemical shifts δisc. Final concentration of 4 mM peptide solutions were attained. NMR experiments for collecting structural constraints were performed at 300 K referenced to external TMS (δ = 0 ppm). The temperature dependence of the amide chemical shifts was observed in the range 300–311K. Deuterium oxide (D_2_O) was purchased from Cambridge Isotope Laboratories (Andover, MA, USA). Mono (1D) and two dimensional (2D) spectra were accumulated with a spectral width of 6000 Hz. 2D experiments DQFCOSY [16], TOCSY [15], ROESY [18] and NOESY [17] were recorded using the States-Haberkorn method. Water suppression was achieved by DPFGSE sequence [47]. TOCSY, NOESY and ROESY spectra were acquired with mixing times of 70, 250 and 150 ms, respectively. Typically, 64 transients of 4K data points were collected for each of the 256 increments; the data were zero filled to 1K in ω1. Squared shifted sine-bell functions were applied in both dimensions prior to Fourier transformation and baseline correction. Data were processed and analyzed using VNMRJ and CARA software [48].

### 3.5. Calculation of 2-HexaPY Ensemble Conformers

Ensemble conformers of 2-HexaPY was calculated by ENSEMBLE software [33,34,35] using the protocol defined by Forman-Kay and co-workers [33]. NMR chemical shifts, distances derived from ROESY experiment and ^3^*J*_HNH__α_ scalar coupling constants were used as experimental constrains. In particular, we used 70 short-range and medium-range NOEs including aromatics and amide protons and we treated them differently based upon their quality. For most NOEs, the two atoms from which they arose were restrained to be within 8 Å of each other. Those NOEs that were identified as being weak or as possibly arising from exchange were restrained to be within 10 Å of each other and were given 10% of the weighting of the stronger NOEs. The residue-specific secondary structure propensity of the ensemble was estimated by STRIDE [42]. Families of structures were extracted from the ensemble of models by superimposing the backbone of residues 3–10 of 2-HexaPY using the program NMRCLUST [43]. The ensemble was visualized and evaluated by using the programs PyMOL [49], MOLMOL [50] and CHIMERA [51].

### 3.6. Diffusion Experiments

The hydrodynamic radius R_h_ for the peptide in water and 8M urea was calculated from the translational diffusion coefficients, D_trans_ by using the Stoke-Einstein equation. PFG diffusion measurements with the PG-SLED (pulse gradient-stimulated echo longitudinal encode-decode) sequence [52] permitted to obtain D_trans_ [53]. Each diffusion data set contained a series of 20 monodimensional ^1^H spectra with gradient strength from 0.5 to 30 G/cm. D_trans_ data were obtained by DOSY package of the VNMRJ software. The hydrodynamic properties of the 2-HexaPY in aqueous solution were also estimated by using HYDROPRO software [37,38,39].

## 4. Conclusions

Mammal and avian prion proteins both contain repeated regions in the N-terminal portion: mammal octarepeats (PHGGGWGQ) exhibit a high number of glycines while chicken hexarepeats (PHNPGY) are characterized by a high number of prolines. The presence of the PXXP motif in the avian prion protein suggests that this region may possess a defined secondary structure. We undertook, by means of CD, NMR and MD techniques, an in-depth structural analysis on a dodecapeptide (2-HexaPY) composed of two chicken hexarepeats. Our results indicate that the 2-HexaPY peptide in solution is in a complex equilibrium between different interconverting conformations. We demonstrate using CD and NMR data that the peptide is not completely random coil but shows a significant propensity to occupy the PPII helical conformation. In order to have a rigorous interpretation of experimental data and to quantify such residual secondary structure we generated, using a combination of experimental NMR data and molecular dynamics, the ensemble of conformers. We demonstrate how each residue of both repeats has a different quantified PPII propensity that shows a periodicity along the sequence. This feature explains the absence of the cooperativity necessary to stabilize a PPII: overall, we estimated for 2-HexaPY peptide a 14% PPII population. Nonetheless, such residual structure can play a role in nucleating local structural transitions as well as modulating intramolecular or intermolecular interactions.

In fact, the residual PPII structure detected for 2-HexaPY peptide suggests a possible stabilization upon interaction with specific binding partners (protein or metal ion), which according to the “conformational selection” theory [54,55], select and stabilize the appropriate conformation.

## Supplementary Materials

Supplementary materials can be accessed at: http://www.mdpi.com/1420-3049/18/9/11467/s1.
