# Supplementary material for: Probing the Residual Structure in Avian Prion Hexarepeats by CD, NMR and MD Techniques
Source: Molecules. 2013 Sep 16;18(9):11467–84. doi: 10.3390/molecules180911467 (PMC6270093; doi:10.3390/molecules180911467)
Supplement: Supplementary file 1 [file molecules-18-11467-s001.pdf]

Supplementary Materials

Table S1. Unambiguous inter-residues and intra-residues NOEs used as structural restraints for the ensemble calculation of 2-HexaPY peptide in H_2O/D_2O (90:10). NOEs were treated as described in the Experimental section.

**PRO 1**	**HA**	**HIS 2**	**H**
HIS 2	HA	ASN 3	H
ASN 3	HA	PRO 4	HD2
ASN 3	HA	PRO 4	HD3
ASN 3	HB2	PRO 4	HD3
PRO 4	HA	GLY 5	H
GLY 5	H	TYR 6	H
TYR 6	HA	PRO 7	HD2
TYR 6	HA	PRO 7	HD3
PRO 7	HA	HIS 8	H
HIS 8	HA	ASN 9	H
HIS 8	HB2	ASN 9	H
HIS 8	HB3	ASN 9	H
ASN 9	HA	PRO 10	HD2
ASN 9	HA	PRO 10	HD3
ASN 9	HB2	PRO 10	HD2
ASN 9	HB3	PRO 10	HD2
PRO 10	HA	GLY 11	H
GLY 11	H	TYR 12	H
PRO 1	HB2	PRO 1	HA
PRO 1	HB3	PRO 1	HA
HIS 2	HA	HIS 2	HB2
HIS 2	HD2	HIS 2	HE1
HIS 2	H	HIS 2	HA
HIS 2	H	HIS 2	HB2
HIS 2	H	HIS 2	HB3
ASN 3	HA	ASN 3	HB2
ASN 3	HA	ASN 3	HB3
ASN 3	H	ASN 3	HA
ASN 3	H	ASN 3	HB2
ASN 3	H	ASN 3	HB3
PRO 4	HB2	PRO 4	HD2
PRO 4	HB2	PRO 4	HD3
PRO 4	HB3	PRO 4	HA
PRO 4	HB3	PRO 4	HB2
PRO 4	HB3	PRO 4	HD2
PRO 4	HB3	PRO 4	HD3
PRO 4	HD2	PRO 4	HA
PRO 4	HD3	PRO 4	HD2
TYR 6	H	TYR 6	HA
TYR 6	H	TYR 6	HB2
TYR 6	H	TYR 6	HB3

Table S1. *Cont.*

**PRO 1**	**HA**	**HIS 2**	**H**
PRO 7	HB3	PRO 7	HA
PRO 7	HB2	PRO 7	HA
PRO 7	HB3	PRO 7	HD3
PRO 7	HD2	PRO 7	HD3
HIS 8	HA	HIS 8	HB2
HIS 8	HA	HIS 8	HD2
HIS 8	HB2	HIS 8	HD2
HIS 8	HB3	HIS 8	HB2
HIS 8	HB3	HIS 8	HD2
HIS 8	HD2	HIS 8	HE1
HIS 8	H	HIS 8	HA
HIS 8	H	HIS 8	HB2
HIS 8	H	HIS 8	HB3
HIS 8	H	HIS 8	HD2
ASN 9	HA	ASN 9	HB2
ASN 9	HA	ASN 9	HB3
ASN 9	H	ASN 9	HA
ASN 9	H	ASN 9	HB2
ASN 9	H	ASN 9	HB3
PRO 10	HB2	PRO 10	HA
PRO 10	HB2	PRO 10	HD3
PRO 10	HB3	PRO 10	HA
PRO 10	HB3	PRO 10	HD2
PRO 10	HB3	PRO 10	HD3
PRO 10	HD2	PRO 10	HB2
GLY 11	H	GLY 11	HA1
GLY 11	H	GLY 11	HA2
TYR 12	HA	TYR 12	HB3
